# The Fulfillment Center Intervention Study: Protocol for a group-randomized control trial of a participatory workplace intervention

**DOI:** 10.1371/journal.pone.0305334

**Published:** 2024-07-18

**Authors:** Erin L. Kelly, Kirsten F. Siebach, Grace DeHorn, Megan Lovejoy

**Affiliations:** 1 MIT Institute for Work and Employment Research and MIT Sloan School of Management, Massachusetts Institute of Technology, Cambridge, MA, United States of America; 2 Bloomberg School of Public Health, John Hopkins University, Baltimore, MD, United States of America; 3 MIT Institute for Work and Employment Research, Massachusetts Institute of Technology, Cambridge, MA, United States of America; 4 T.H. Chan School of Public Health and Harvard Center for Population and Development Studies, Harvard University, Cambridge, MA, United States of America; International University - Vietnam National University Ho Chi Minh City, VIET NAM

## Abstract

Warehousing and storage is an economically vital industry, with 1.2 million workers in 2020. The Fulfillment Center Intervention Study focuses on workers in fulfillment centers in the e-commerce segment of this industry. Fulfillment centers are a growing yet understudied work environment which provides a unique setting to further examine how working conditions and worker voice influence health. The Study involves a group-randomized controlled trial comparing participants in worksites randomized to launch the participatory intervention (Health and Well-Being Committees, or HaWCs) with participants working for the same firm in control sites. HaWCs serve as a new formal voice channel where a small group of frontline workers and supervisors solicit workers’ concerns and ideas about safety (e.g., physical hazards), the psychosocial environment (e.g., how workers feel about their treatment at work), and work organization (e.g., workflow, training opportunities, scheduling) and then develop and implement improvement projects in response. The primary objectives of the study are to evaluate the efficacy of the HaWC intervention and its effect on mental health outcomes and changes in the conditions of work within fulfillment centers, and to conduct a process evaluation of key contextual factors that support effective intervention implementation and sustained engagement. To our knowledge, this will be the first trial of a participatory intervention within a fulfillment center setting. Anticipated challenges include competing demands and company initiatives that may limit management support and high turnover. Should the intervention be shown to be feasible, the outcomes from this study will inform future randomized controlled trials of participatory interventions.

**Trial registration:** This trial is registered with the ClinicalTrials.gov registry (NCT05199415) and approved by the Institutional Review Board (IRB) at the Massachusetts Institute of Technology (Protocol: 200800024).

## Introduction

The work environment is an important social determinant of mental and physical health. Poor working conditions, including high job strain [1, 2], non-standard work schedules, long hours [3, 4], limited schedule control [5, 6], and limited voice [7] (i.e., workers’ opportunities to share ideas and concerns about their work environment) are associated with higher risk of injury and low job satisfaction [8] as well as common mental disorders, such as anxiety and depression, and poor psychological well-being [9]. Poor mental health can have a negative impact on health across the life course, including increased disability and higher risk of chronic diseases and premature mortality risk [10, 11], whereas positive psychological well-being predicts healthier aging and greater longevity [12]. Healthy and supportive work environments are not only important for the individual health of workers, but also for the economy more broadly. A 2022 report estimated that poor mental health in the U.S. workforce costs the economy $47.6 billion each year in lost productivity [13].

The Warehousing and Storage industry provides a unique setting to further examine how working conditions and specifically worker voice influence health. This industry has experienced rapid growth in recent years; employment has nearly doubled since 2000 to 1.2 million workers in 2020 [14]. Much of this growth has been fueled by the rise of e-commerce (i.e., buying and selling goods on the Internet), a segment of the warehousing industry that relies on work done in fulfillment centers; data on the fulfillment center workforce specifically is more limited. Fulfillment center sales rose by 52%, contributing to a 37% increase in employment in the broader warehousing industry, between 2014 and 2017 [15]. Black and Latino workers are overrepresented in fulfillment centers, as compared to the overall U.S. labor force (Black workers: 25% vs. 12.3%; Latino workers: 35% vs. 18%) [15]. The racial and ethnic diversity of this workforce both reflects the future of American demographics and exposes workers already facing systemic health inequities to difficult working conditions. Given this growth and composition, it is critical to investigate factors that influence worker health in fulfillment centers.

In the midst of this growth, and especially in light of the increased demand absorbed by these workers during the COVID-19 pandemic, it is important to understand how the conditions of work in fulfillment centers affect workers’ health. Fulfillment center workers face conditions that have been demonstrated to affect health in other industries, including high job strain (high job demands and low job control [1]), non-standard schedules, long hours [3], and limited schedule control [5] that affects sleep duration [16] and increases work-family conflict [6, 17, 18]. These work conditions also increase the risk for common mental disorders, poor psychological well-being, and injury [9]. Data on fulfillment centers are limited, but the warehousing, transportation, and utilities sector has among the highest prevalence of low job control, anxiety, stress, and neurotic disorders [19]. Risks of injury and illness are high, with an incidence rate of 5.1 for warehousing and storage compared to 3.1 for all industries and twice the rate for cases with days away from work [20].

Fulfillment center workers have few opportunities for exercising “voice,” a key component of our proposed intervention. Perceived voice—the ability to express opinions to improve work conditions [21] predicts job satisfaction and contentment [7]. Unions have historically provided a voice mechanism through which workers could share their concerns and influence how work is accomplished, however union representation within fulfillment centers is sparse [7, 12]. Filling the gap in research as it pertains to the health impacts of working conditions within fulfillment centers, including voice as another measure of the psychosocial work environment, can provide critical guidance for the development of strategies that will improve population more health broadly.

The Total Worker Health (TWH) approach, developed by the National Institute of Occupational Safety and Health (NIOSH) aims to improve the work environment and promote work-related health and well-being through the integrated redesign of workplace policies, programs, and practices. TWH approach uses an integrated approach to improving well-being by combining a traditional concern with safety and protection from injury with a focus on conditions such as workplace psychosocial supports and the organization of work which are related to health promotion and other components of health and well-being more broadly [22]. Interventions employing this approach have demonstrated effectiveness in improving risk factors for injuries and illness across a range of industries, including manufacturing, services, health care, construction, telecommunications, and transportation [23, 24]. In their review of TWH intervention studies, Anger and colleagues [23] found that all but one of the included studies demonstrated statistically significant improvements on study outcomes. The authors concluded that TWH-oriented interventions that address both injuries and broader wellness and well-being outcomes can improve workforce health effectively and more rapidly than traditional programs focused exclusively on occupational safety and health.

Our study responds to a growing interest in participatory interventions, which create structured opportunities to exercise voice and involve workers in problem solving [25–28]. According to Nielsen and Randall [29], a participatory workplace intervention includes involving workers in intervention design, identifying areas of improvement, developing action plans, implementing action plans, and evaluating the results of the intervention [29]. From the perspective of voice research, participatory interventions give workers the opportunity to influence how they do their work (i.e., often aim to increase job control) but also provide opportunities to share ideas, concerns, and expertise more broadly. Unlike more traditional voice mechanisms such as unions, participatory interventions are unique in that they involve input from both management and workers seeking mutually beneficial solutions. Participatory interventions can therefore be understood as a new voice mechanism, a new channel for workers’ input into the organizational environment. On-going interventions, in particular, provide repeated opportunities to share concerns and ideas; those who are most directly involved also have the opportunity to contribute to planning and implementing changes in their workplace.

Participatory workplace interventions have demonstrated positive effects on worker well-being [30]. Previous research has shown that worker participation is an essential aspect of effective organizational interventions [31] and that worker participation can improve health and safety [32, 33]. Cross-sectional research finds that worker involvement is associated with higher job satisfaction, less fatigue, and lower odds of injury [34]. The adoption of safety committees [35] with a broader scope and higher worker involvement is associated with fewer injuries [36]. Despite these clear potential benefits for worker well-being, the integrated and participatory TWH approach has yet to be studied in the fulfillment center work environment.

The aim of this paper is to present the study protocol of a cluster randomized trial designed to evaluate the effectiveness of a participatory intervention of Health and Well-being Committees (HaWCs) in fulfillment centers consistent with the TWH model. The primary study hypotheses were that workers in fulfillment centers randomized to receive this 12-month intervention would have reduced psychological distress and improved psychological well-being compared to those individuals in control sites. The process evaluation will document how the intervention is implemented across each intervention site.

## Methods

The description of the study follows the CONSORT statement, with the extension for a cluster randomized trial [37].

### Study design

The study is a two-arm cluster randomized-controlled trial. The units of analysis are individuals within the cluster of fulfillment centers. There are three measurement time points: baseline measured prior to randomization (T1), 6 months after baseline (T2), and 12 months after baseline (T3). Process evaluation data will be collected for approximately 30 months, from baseline through 18 months after the T3 survey. The study has been registered with the ClinicalTrials.gov registry (NCT05199415) and approved by the Institutional Review Board (IRB) at the Massachusetts Institute of Technology (Protocol: 200800024). Any changes to the protocol will be approved by the IRB.

### Study setting, population, and recruitment

The study utilizes an established research partnership with the supply chain division of a mid-sized, non-unionized U.S. based retailer, heretofore referred to as “Sigma.” Sigma employs approximately 4,800 non-supervisory employees in 24 fulfillment centers which are geographically dispersed across the United States. Within a given fulfillment center, the main job role is “picking,” whereby workers pick products, box them, and ship them based on a customer’s order. Each fulfillment center utilizes one of three technology systems to facilitate this work process: conveyors, carts, or robotics. Workers’ productivity, or how many orders a worker fulfills, as well as a workers’ “time off task,” is monitored via additional technology.

Formative research and intervention development occurred in 2019 and 2020 and baseline recruitment of fulfillment centers began in July 2021. Given research team staffing constraints and Sigma’s interest in avoiding data collection during periods of high demand, there was a sequential roll-out of the intervention in three groups of fulfillment centers between July 2021 and July 2022. See page 8 for additional details on participant recruitment.

#### Inclusion criteria

All fulfillment centers (N = 24) are assessed for eligibility for study inclusion.

#### Exclusion criteria

**Fig 1:**
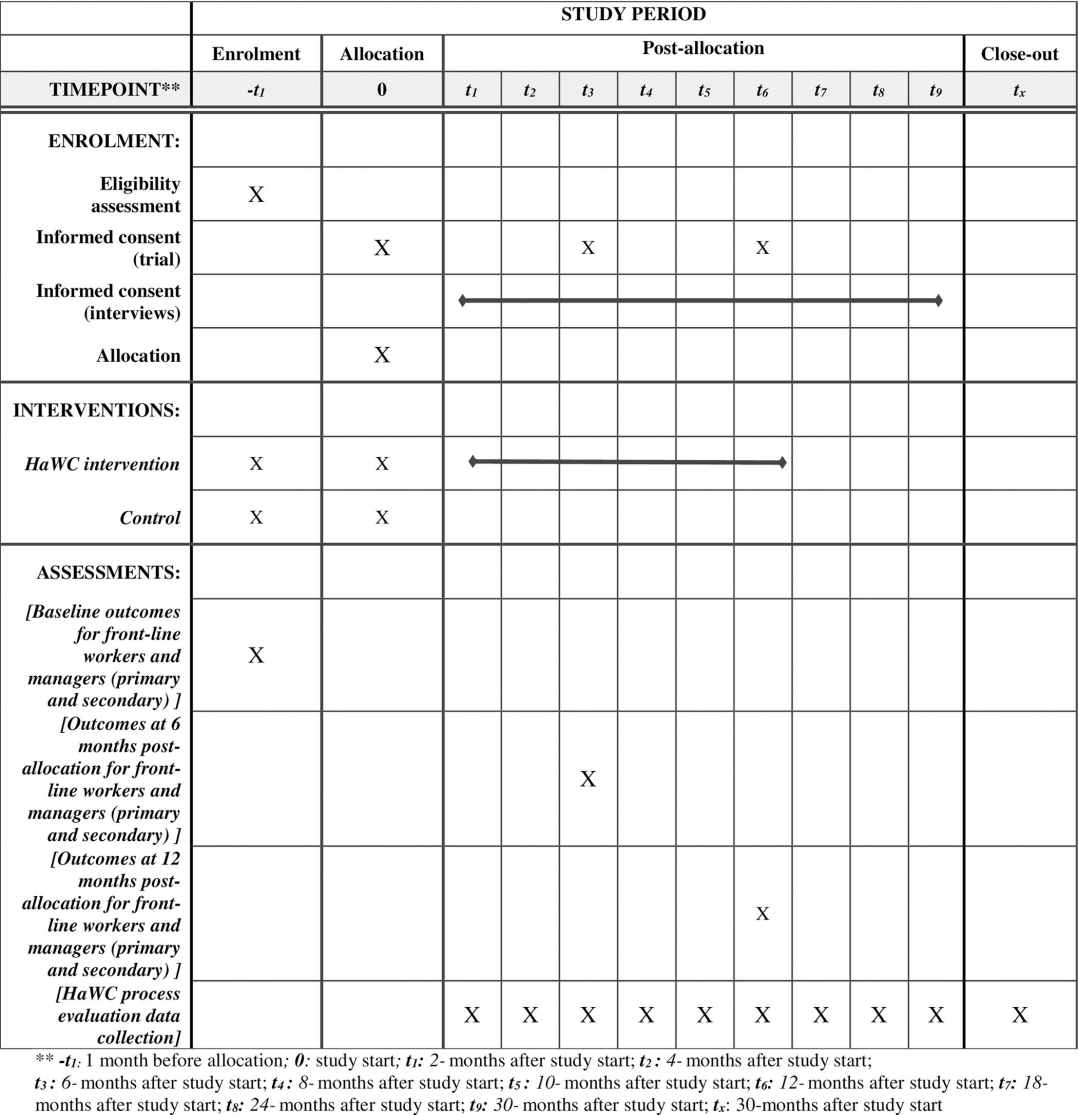
Schedule of enrolment, intervention, and assessments.

Fulfillment centers are excluded from randomization if: there was a previously established union in the fulfillment center (N = 1), there are < 15 employees at the site (N = 1), the site served as the pilot site (N = 1), the site shuts down during the study period (N = 1), or the site experiences a major change in leadership prior to or at the time of the baseline survey (N = 4). The research team and Sigma agreed to exclude the unionized site due to its distinctive nature and because the vast majority of sites did not have any unionized workers. Future research could explore how this participatory intervention can be implemented within a unionized context. See Fig 1 for the schedule of enrolment, intervention, and assessment.

### Randomization

Following baseline survey administration, fulfillment centers are matched based on the main picking technology (conveyors, carts, or robotics) and size (small, medium, large, or very large) based on the number of workers within the fulfillment center. Within each technology and size group, each fulfillment center is randomized to either receive the intervention or to maintain operations as usual. Randomization occurred by randomly assigning numbers to be intervention or control and then randomly assigning them to study buildings. Site managers are then notified of their fulfillment center’s treatment assignment. Given the geographic distance between fulfillment centers, the likelihood of contamination between sites was considered minimal.

### Blinding

Participants undergoing baseline measurement are unaware of arm allocation as randomization occurs after baseline. Due to the nature of the intervention, it will not be possible to blind participants throughout the data collection process. However, all recruitment materials will describe the study goals quite broadly *(e*.*g*., “The purpose of the study is to understand what it’s like to work at a fulfillment center in general and to understand how changes in the workplace affect your experience working at [Sigma]”) and will not mention the intervention. Additionally, those conducting final data analyses will be blinded to group allocation.

### Intervention

**Fig 2:**
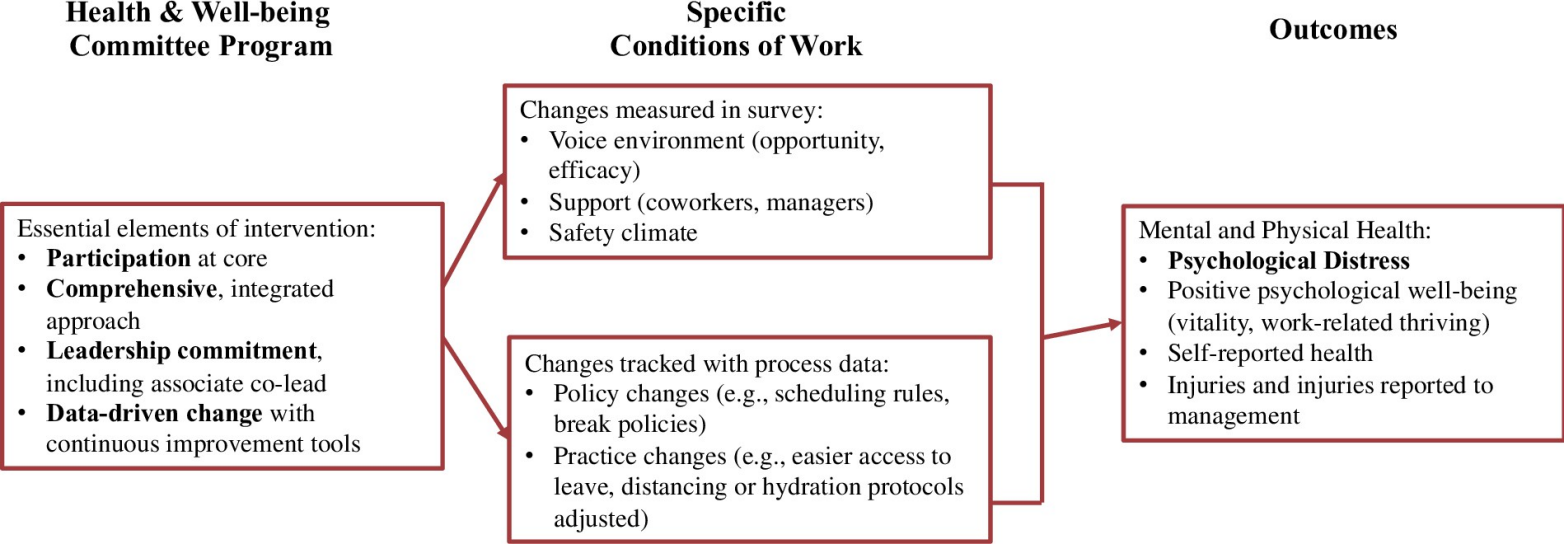
Health and Well-Being Committee participatory intervention model.

The research team developed a new intervention, Health and Well-Being Committees (HaWCs), which is informed by existing workplace participatory programs and by best practices for engaging workers in safety initiatives and continuous improvement strategies, e.g., problem identification and definition, solution brainstorming, and solution implementation and testing [35, 38, 39]. The intervention was piloted in a fulfillment center between September 2020 and July 2021, after which the intervention was refined and finalized based on input from the pilot site. Fig 2 presents the intervention model with expected changes.

HaWCs will be composed of 10–14 frontline workers and supervisors and led by a “co-lead” pair, comprised of one frontline worker and one supervisor. The dual management-worker leadership structure is designed to facilitate a joint problem-solving orientation with a focus on multiple stakeholder interests and mutual gain. Members of the HaWC are expected to be volunteers and to represent different departments, tenure, and social identities (e.g., gender and race). HaWC meetings will occur when associates and supervisors are “on the clock” and compensated for their time. HaWCs are designed to be a new formal voice channel where workers can submit concerns about the workplace environment pertaining to safety (e.g., physical hazards), the psychosocial environment (e.g., how workers feel about their treatment at work), and work organization (e.g., workflow, training opportunities, scheduling). HaWCs then will be charged with prioritizing concerns, designing, coordinating, and implementing solutions, and tracking outcomes.

Following randomization, managers in intervention sites will meet with the research team to plan the intervention launch, identify possible co-leads, and plan recruitment of HaWC members. Co-leads will then participate in a multi-session training delivered by the research team where they will be trained in the continuous improvement strategies and facilitation skills.

The intervention is comprised of four distinct stages designed to provide scaffolded support from the research team to the co-leads. The intervention stages include: the Start-up Phase, the Transition Phase, the Sustainability Phase, and the Maintenance Phase. The Start-up Phase (months 0–2 and meetings 1–4) consists of biweekly HaWC meetings during which the research team is highly involved in meeting planning, joint facilitation, and agenda setting. In the Transition Phase (months 2–4 and meetings 5–8), the research team continues to provide support while handing over responsibilities for meeting planning and facilitation to the co-leads. During the Sustainability Phase (months 4–12), the co-leads are expected to fully run HaWC activities but consult with the research team approximately once every other month and on an informal basis as needed. The Maintenance Phase (months 12–30) begins after the quantitative evaluative period (T3) and consists of less frequent, but regular interaction between the research team and HaWC sites (approximately every 6 months) in order to evaluate whether and how the intervention is sustained.

The Project Improvement Cycle begins with bi-weekly one-hour HaWC meeting. The cycle, shown in Fig 2, provides structure for meeting discussions. During these meetings, committee members hear suggestions from each other as well as suggestions that coworkers share via comment boxes or discuss directly with a HaWC member, prioritize work concerns, conduct a root cause analysis of the concern if deemed useful, brainstorm solutions, and develop and implement action plans. Following the implementation of an action plan, the improvement cycle will begin anew. The improvement cycle also provides flexibility for HaWCs to spend more or less time on a particular step, depending on the topic, allowing for a customized approach for addressing the needs of each particular site.

A phased rollout was planned to be responsive to the firm’s busy periods and the research team’s capacity. Additionally, significant delays related to operational and staffing challenges in the wake of the COVID-19 slowed the timeline for the study launch. The intervention is still ongoing.

### Data collection and measures

Table 1 provides an overview of data collection activities, aligned with overall study objectives.

**Table 1:** Overview of study objectives and supporting research methods.

Study Objective	Supporting Research Method
Evaluate the efficacy of the HaWC intervention and its effect on health outcomes and changes in the conditions of work	Self-reported surveys from workers in HaWC and control sites (T1, T2, T3)
	Administrative data collection for all sites, including turnover rates, other changes in workplace policies or practices
Conduct a process evaluation of key contextual factors that support effective and integrated intervention implementation and sustained engagement	Tracking meetings and projects in HaWC sites (detailed tracking through T3, summaries after)
	Co-lead interviews in HaWC sites (every 6 months)
	Manager interviews in HaWC and control sites (T2, T3)
	Focus Groups in HaWC sites (T3)

Survey measurement occurs at three time points: baseline (T1), 6 months (T2), and 12 months (T3). At each time point, all workers currently working in the fulfillment center are presented with an individualized invitation letter to complete a web-based survey via Qualtrics. Participants can access the questionnaire using a cell phone, research-team provided tablets, or desktop computers at the worksite. All participants will provide informed consent prior to participation. All participants are given the opportunity to participate in the survey while on-the-clock, but also have the option to participate outside of work. To promote participation, all individuals who complete the questionnaire are given a small incentive at each wave. Those who complete the questionnaire are also eligible to receive site-level raffles. In addition to the self-reported questionnaires, administrative data on worker characteristics and injuries and fulfillment centers-level characteristics are collected from Sigma databases.

Given the low-risk nature of the study, adverse events are unexpected. However, to ensure safety of study participants, the research team will proactively ask building managers whether concerns have arisen about the study. Should any concerns arise, participants will be able to contact the IRB and Human Resources department at Sigma.

### Outcome measures

Table 2 presents a description of the outcomes measured in the study. All outcomes are measured at each of the three timepoints in both treatment and control sites.

**Table 2:** Overview of outcome measures.

Outcome	Source	Description
**Primary**		
Psychological distress	Questionnaire	Six-item short form of the Kessler distress scale [40]. Assesses the frequency of symptoms of psychological distress experienced in the last month.
**Secondary**		
Psychological well-being	Questionnaire	Six items assessing emotional vitality, sense of positive well-being, and emotional self-control in the last month [41].
Work-related well-being	Questionnaire	Burnout: Two-item Maslach burnout scale [42]. Job satisfaction: One item rating satisfaction with the job from 0–10. Engagement: Two items assessing whether a worker’s mind is focused on their job and whether their job is boring.
Psychosocial work environment	Questionnaire	Nine items from the Thriving from Work Questionnaire that assess perceptions of fair treatment, respect, belonging, purpose, support, work-life balance, fair pay, and job security.
Voice environment	Questionnaire	Worker voice: Three items reporting on whether workers know who to talk to when they have an issue at work, whether they can make suggestions without getting into trouble, and whether leadership takes their concerns seriously.
Conditions of work	Questionnaire	Job demands and control: Six items on decision authority, skill discretion, and psychological job demands. Safety climate: Six item safety climate scale and additional safety climate items specific to the COVID-19 pandemic.
Self-reported health	Questionnaire	General Health: A single general item from SF-36. Musculoskeletal pain: A single modified item from the NordicQ. Sleep: A single item assessing sleep duration in the last 30 days from the Pittsburgh Sleep Quality Index.
Injuries	Questionnaire Administrative Data	Questionnaire: Three items asking whether an injury was experienced, whether it was reported, and the reason for not reporting. Administrative data: Captures total injuries reported to Occupational Safety and Health Administration and number of recordable injuries per 100 workers, by site.

#### Primary outcome

The primary outcome is psychological distress, measured with the 6-item short form of the Kessler distress scale (K6) [40]. The K6 scale has been used widely in workplace studies as a short screener for discriminating between those who do and do not meet diagnostic criteria for a mental disorder. This scale has excellent internal consistency and reliability (Cronbach’s alpha = 0.89) [40]. Participants are asked the frequency of non-specific symptoms of psychological distress (feeling “nervous,” “hopeless,”, “restless or fidgety,” “so depressed that nothing could cheer you up,” “that everything was an effort,” and “worthless”) in the month prior to data collection. Response items are on a 5-point scale, ranging from (0) “none of the time” to (4) “all of the time”. Items are then summed to create an overall score between 0 and 24, with higher scores indicating higher levels of psychological distress and a score ≥13 suggesting severe mental illness [43].

#### Secondary outcomes

In addition to the primary outcome, the study will analyze the effect of treatment on several secondary outcomes (see Table 1). Psychological well-being is measured with a six-item scale comprised of three, two-item subscales assessing emotional vitality (e.g., waking up fresh and rested, how much energy, and vitality the participant had), sense of positive well-being (e.g., how happy, satisfied, or pleased the participant has been in their personal life, whether daily life has been full of things that were interesting), and emotional self-control (e.g., whether the participant has been in firm control of their behavior, thoughts, emotions, or feelings, and whether the participant has been feeling emotionally stable and sure of themselves) [41]. Responses options are on a 6-point scale that ranges from (0) “not at all” to (5) “a lot or all of the time”, though the item assessing energy and vitality ranges from (0) “no energy at all, listless” to (10) “very energetic, dynamic”.

Work-related well-being is measured with three related constructs. Burnout is measured with two items that assess emotional exhaustion (e.g., “I feel burned out from my work.”) and depersonalization (e.g., “I have become more uncaring toward people since I took this job.”). These two items are taken from the Maslach Burnout Inventory [42] and have been validated as a measure of burnout [44]. Job satisfaction is a one item rating satisfaction with one’s job from 0 to 10. Engagement is measured on a Never-Always scale by focus on work (e.g., “At work, my mind is focused on my job.”),

Psychosocial work environment is assessed with 9 items from the Thriving from Work Questionnaire [45]. These items capture the perception of fair treatment, belonging, purpose, support, work-life balance, and fair pay. Voice environment was assessed with three items. Workers report on whether they know who to talk to when they have an issue at work, whether they can make suggestions without getting into trouble, and whether leadership takes their concerns seriously on a 6-point scale from (1) “never” to (5) “always or almost always” [46].

Other conditions of work are assessed with questions on job control and demands and safety climate. Job control and demands are measure with six items from the Job Content Questionnaire. Participants report on decision authority (2 items), skill discretion (2-items), and psychological job demands (2 items) on a 5-point scale from (1) “strongly disagree” to (5) “strongly agree” [47]. Safety climate (e.g., “I am provided with what I need to work safely,” and “Safety of workers is a big priority with supervisors and managers.”) is measured with 6-items assessed on a 5-point scale from (1) “strongly disagree” to (5) “strongly agree” [48–50]. As the study data collect spans the COVID-19 pandemic, a 5-item safety climate scale specific to COVID-19 is also included [51].

Self-reported health is measured with three separate items assessing different domains. General health is assessed using one item from the 36-item short form (SF-36) [52], which asks participants to rate their health from (1) “poor” to (5) “excellent”. Musculoskeletal pain is measured with a single item from the NordicQ [53]. Participants are asked whether they have experienced pain or aching (yes/no) in different body regions (e.g., back, shoulders/neck, hands/wrists/arms, and legs/knees/feet), and if yes, they are asked to rate the pain from (1) “mild” to (3) “severe”. One item from the Pittsburgh Sleep Quality Index [54] asks participants to report sleep duration in the last 30 days.

Injuries are assessed using both the questionnaire and administrative data. In the questionnaire, workers are asked whether they experienced an injury in the last 12 months, whether they reported the injury to management, and if it was not reported, the reason for not doing so. Administrative data captures the total injuries reported by the firm to the Occupational Safety and Health Administration (OSHA), as well as the Total Recordable Injury Rate (TRIR) by site. OSHA defines a recordable injury as any work-related fatality or injury that results in loss of consciousness, days away from work, restricted work, job transfer, or requires medical treatment beyond first aid [55].

### Covariates

Worker characteristics, including age, gender, race and Hispanicity are captured with administrative data and linked to the survey data. Additional personal characteristics (marital status, children at home) are self-reported in the questionnaire. Tenure with the firm, department, hourly wage, part-time vs full-time job, and shift are included through administrative data.

### Data management and sharing

All survey data will be collected using the Qualtrics platform. Data will be downloaded from Qualtrics frequently during the survey period and kept on a secure server only accessible to the data team. Though the survey will collect identifiable information for the purposes of incentive distribution, participants’ data will deidentified immediately after their incentive is sent. Given the low-risk nature of this study, a data monitoring committee is not needed. Survey data will be available through a restricted access process after the acceptance for publication of the main findings. Those who wish to use the data will need to complete an application with their affiliate institution’s IRB and provide a brief description of their project. The data sharing agreement will state that a) the data access will last for one year, with the opportunity to extend on a case-by-case basis, b) those who use the data will do so only for research or statistical purposes and not for investigation of the research subjects, c) published paper will include the appropriate acknowledgement as stated in the data documentation, and d) the data will not be distributed to anyone other than the approved researcher.

### Data analysis

Preliminary data analyses will include descriptive statistics by study arm (intervention or control) to summarize characteristics of workers and fulfillment centers. In the primary analysis, the effect of the intervention on the primary and secondary outcomes will be investigated using intention-to-treat (ITT) analyses with multilevel linear mixed models for continuous outcomes and generalized mixed effect models for binary outcomes. Models will include random intercepts for clusters and workers within each site, a fixed effect for each stratum, a fixed effect for each follow-up time point (T1 and T2), and an interaction effect between intervention status and follow-up time. The interaction between intervention status and follow-up time will explicitly test for intervention effects. All models will be adjusted for covariates, such as age, race, gender, calendar time, and site level policy changes, as appropriate. While the primary analysis will be ITT, exploratory analysis will consider whether number of HaWC action projects and a coded typology of projects (e.g., safety focused vs. operational vs. focused on the broader psychosocial environment) affects the impact of the intervention.

As a secondary analysis, another model will be fit among workers who have participated in baseline and at least one follow-up survey to directly control for baseline values of outcomes of interest. Both the primary and secondary analysis will include calendar time to account for changes in the larger environment, such as seasonal fluctuations in demand, seasonal changes in distress, and pandemic variations in COVID-19 death rates. Sensitivity analysis will include an assumption of non-ignorable missingness based on an imputation approach developed specifically for longitudinal data [56]. Estimates from mixed-model regressions will be considered valid when missingness across assessment time-points is missing at random (MAR), assessed using logistic regressions for response bias [57, 58].

Beyond the primary and secondary analysis, intervention effects will be measured with voice environment as a primary mediator. Both the difference and product method will be used in mediation analysis; the latter can incorporate possible mediator-intervention interaction. The results of these models will be used to obtain indirect effects of the intervention on outcomes of interest. Process data will also be used to measure intervention effects that reflect differential intervention implementation.

### Power calculations

Statistical power was calculated using a closed-form sample size formula specifically for longitudinal cRCTs with attrition, which accounts for intracluster correlation (ICC), repeated measures on the same workers, and expected attrition due to turnover [59]. By sampling 21 clusters (10 treatment and 11 control), with an average of 200 per cluster, the study population was estimated to be a total of 4200, with 1100 employees in the intervention group and the control group respectively. Based on administrative data which showed a 6-month turnover rate of 28%, attrition between waves was estimated to be A = 0.3. As there are no previous intervention studies in this workforce, an ICC of 0.01 was assumed based on a previous study of a participatory intervention in a low-wage and racially diverse workforce with the same primary outcome of psychological distress measured with the K6 scale [60]. With an assumed response rate of 66%, there will be 90% power to detect an effect size of (Δ) 0.13 standard deviations (SDs) and a mean difference of 0.56 in psychological distress score and 80% power to detect Δ = 0.12 SDs and a mean difference of 0.51. With an assumed response rate of 44%, there will be 90% power to detect an effect size of Δ = 0.15 SDs, or mean difference of 0.56, and 80% power to detect Δ = 0.15 SDs and a mean difference of 0.51. The fixed number of clusters in each power-calculation scenario leads to a smaller increase in power and precision with an increased response rate than if the study were randomized at the cluster level.

### Process evaluation

**Fig 3:**
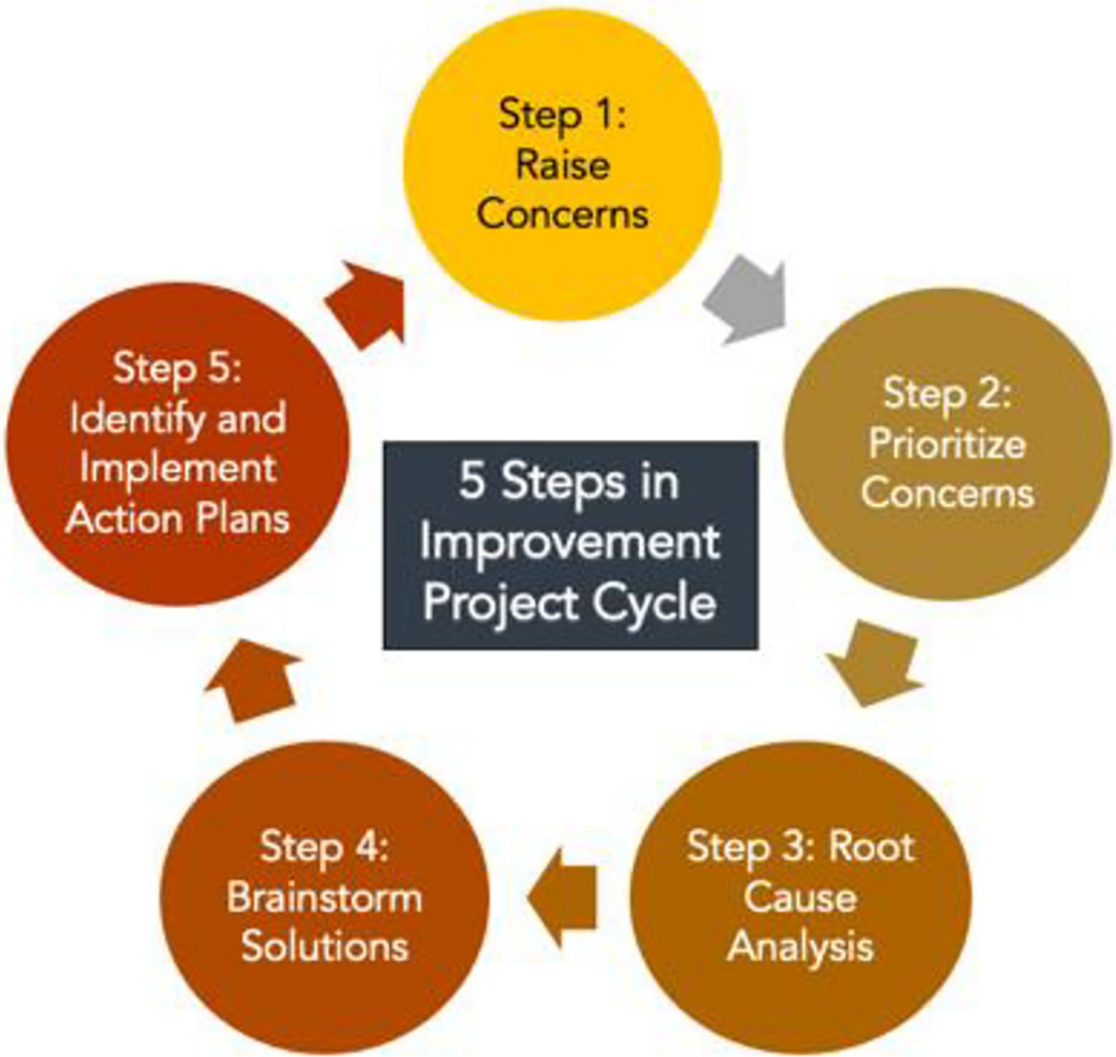
The Project Improvement Cycle.

In addition to the cluster randomized trial, the study will involve a process evaluation to examine intervention fidelity and investigate the dynamic of organizational change. Robust process analysis is particularly important for participatory interventions such as the HaWC program because the targeted changes will vary across sites. Specifically, the process evaluation aims to answer the following research questions: (1) What organizational and contextual factors (e.g., institutionalized power differentials, social identities, and group processes) shape the scope and type of action plans that HaWCs prioritize and adopt? (2) What barriers or challenges do participants face in implementing the intervention and what supports the effective resolution of those difficulties? (3) What factors facilitate sustained engagement once the research team is less involved, i.e., during the maintenance phase of the intervention? To answer these questions, data will be collected at regular intervals from various sources in the intervention sites, including HaWC members (focus groups), HaWC co-leads (repeated interviews), and fulfillment center managers (repeated interviews) (Fig 3). Additionally, the research team will take detailed field notes while observing the HaWC meetings and following interviews. Interview topics include experiences with HaWC processes, HaWC adaptations, barriers to implementation, and any site-specific context that may influence the intervention. Managers at control sites are interviewed at 6, 12 and 30 months (in parallel with manager interviews in treatment sites) to assess any changes in fulfillment center technologies, policies, and practices.

The estimated time per data collection activity is 30–45 minutes for interviews and 60 minutes per focus group. These process data will support case analyses, cross-case comparisons, and mixed methods intervention evaluation. We will pursue an abductive analysis oriented to theory construction in which we focus on what is surprising, given existing theories of participatory workplace initiatives, rather than develop conclusions in a purely inductive process [61]. Coding will be derived both deductively from the existing literature and inductively with the construction of additional categories as new themes emerge [62, 63]. Qualitative analysis will proceed in an iterative process of ongoing data collection, analysis and writing. Data analysis will be conducted using the qualitative software program, Atlas.ti.

## Discussion

This paper presents the design and protocol of a cluster-randomized control trial aiming to improve psychological distress and well-being. The participatory intervention is designed to provide a channel for workers to voice their concerns, implement solutions, and track workplace changes that matter to them. This research provides insight into an understudied and diverse population of fulfillment center workers and will support new analysis of the potential health-promoting effects of worker voice, which receives arguably less attention than other work conditions specific to the design of tasks or expression of support by managers. The research team’s access to the fulfillment centers through site visits and their involvement in the implementation of the HaWCs is a unique opportunity for the team to discover factors that benefit and hinder the implementation of this innovative intervention. The findings from this study will deepen the scholarly understanding of participatory workplace interventions and their effects on worker health and well-being, while also establishing new evidence about feasible changes to support workers in this important and growing industry.

### Strengths

The research team expects the strong causal design, multi-method data, and participatory intervention to provide critical information on the feasibility and efficacy of Health and Well-Being Committees in the important context of fulfillment centers. Studying frontline workers in the warehousing and storage industry is valuable not only because this is a rapidly growing yet understudied sector, but also because research on interventions to improve well-being in low-wage workers in general, is extremely limited. ^24^

Another innovation is the combination of a mental health primary outcome (psychological distress) with analysis of potential impact on positive psychological well-being (emotional vitality). With a strong causal design that utilizes a randomized field trial, the project will provide an evidence base for evaluating the efficacy of a participatory intervention not only to reduce psychological distress, but also to promote well-being, and to improve work conditions among fulfillment center workers. Additionally, the use of a randomized cluster trial provides the opportunity to isolate the intervention from other changes in Sigma’s work environment, including COVID-19 related confounders, and organizational policy changes that may occur. A pre- and post-test study would not be sufficient to tease out these confounders, but the trial allows the team to isolate the treatment’s impact.

The combination of randomized experimental methods with multi-methods process approaches is also important for advancing the field. Few studies thoroughly examine how and why workplace interventions succeed or fail to achieve their intended effects, despite calls to “open the black box” of organizational interventions [64, 65]. Adopting a realist evaluation perspective, we recognize the complexity behind various mechanisms within a work environment that must function together to establish a successful intervention [66]. For this reason, our research team will utilize multi-methods process data to provide critical insights into how, why, and in which contexts interventions such as these may be effective, as well as what facilitates or hinders their implementation and sustainability over time. Additionally, a focus on identifying the scope and content of workplace changes prioritized by HaWC teams (the first process evaluation question) promises to fill a gap in our understanding of how TWH interventions achieve integration across safety concerns, psychosocial support, and the organization of work, and how different organizational actors (i.e., managers and frontline workers) come together to prioritize and implement an agenda for workplace changes.

The study’s coverage of an extended implementation period is another strength, allowing the team to examine the key question of how engagement in the participatory intervention can be maintained over a prolonged period or how they may evolve within a given workplace. Little is known about how intervention engagement can be sustained over time [67], despite the importance of program maintenance for intervention effectiveness.

Participatory processes have been found to be a key element of effective organizational interventions targeting well-being [31]. Our study responds to a growing interest in participatory interventions, creating structured opportunities for workers to exercise voice and be involved in problem solving. With a strong causal design, the use of multi-method data, and the implementation of a participatory intervention, we expect to obtain exciting results that help to address poor mental health and well-being in the understudied industry of warehousing.

### Limitations

While the design of the study is strong, there are anticipated risks to data collection and implementation, in addition to the possibility of limited efficacy of the intervention in this work environment. Concerning data collection, we must establish a strong relationship and build trust with hourly workers in order to obtain the desired data. Due to budgetary constraints and firm concerns with safety of researchers on site, travel is likely to be limited and researchers’ inability to be on-site at the fulfillment centers during the initial launch of the surveys may impact survey response rates. Moreover, Sigma’s own data collection (e.g., company engagement survey) may compete or overlap with the study’s surveys, resulting in survey burnout among frontline workers. The project would benefit from a good response rate, but these are difficult to achieve even with the integration of compelling recruitment materials and the offer of individual incentives. As temporary and subcontractor labor is common in fulfillment centers through the year [68, 69], the study may include temporary workers. However, their participation may be especially hard to elicit given that they may be more detached from the company and managers may not encourage them to participate. High turnover within this workforce may further complicate data collection and analysis, although some models described above do not require repeated measures.

For the implementation of the intervention to move forward successfully, sustained buy-in from Sigma’s leadership team is crucial. Despite having good executive support, formative research has shown that managers within each building operate with a large amount of autonomy. It is plausible that fulfillment center managers could make decisions about the HaWC that impact the continuation of the committees within specific fulfillment centers. The fast-paced environment, and management’s imperative to meet short-term production goals (especially during times of high building volume) may conflict with HaWC program goals of meeting regularly and implementing projects not directly related to production but rather to employee well-being.

The research team must also rely on the fulfillment center managers to help identify the co-leads for the HaWCs, which likely means the inclusion of individuals who have already been labeled as leaders (from the perspective of management) within the fulfillment center. The addition of HaWC responsibilities to these already high-performing workers could result in overload and/or encourage the promotion of these associates to other positions. Turnover in the associate and manager co-leads could challenge the sustainability of the committee, result in the turnover of other members within the committee, and limit other workers’ confidence in sharing concerns with the HaWC. The pandemic may also require that intervention meetings and training be held virtually, which could inhibit the ability to build strong relationships between the research team and the HaWC co-leads and members.

Given intervention goals of supporting employee voice, the fulfillment center’s high-turnover environment will likely pose challenges to the efficacy of the HaWC. Other workers must know and trust the HaWC members in order to share their concerns and support changes related to HaWC projects. High turnover could dilute the impact of the HaWC, since new co-workers may not know about, utilize, or trust the committee’s process. Additionally, high turnover exacerbates productivity pressures that may make managers less likely to support HaWC meetings and projects, as noted above.

### Impact of results

Should workers in fulfillment centers who are randomized to receive the intervention report reduced psychological distress and improved psychological well-being, the project will provide a pathway within the warehousing industry to address longstanding challenges of poor mental and physical health among workers [1–5]. The project will also contribute to scholarly understanding of an understudied vulnerable workforce, one that is diverse by race, ethnicity, nativity, age, and gender [15]. The study works to uplift worker voice, which has historically received less attention than attempts to modify job control, demands, support, and other work conditions. There is also a continued need for evidence-based interventions that embrace a social determinants of health approach and aims to change conditions of work rather than focusing on individual behavior change through wellness initiatives or health promotion [70].

With virtually no studies that identify successful interventions to reduce risks for this population, this project could push the field forward by integrating immediate safety concerns with broader efforts to change work conditions and by adapting process improvement methods common in management research to the goals of improving workers’ health and well-being [28]. The study can produce exciting new data on fulfillment center workers’ health and well-being and the efficacy of this innovative participatory intervention. This will open important avenues for future research, including analysis on enterprise or organizational outcomes, such as absenteeism, turnover rates, and productivity. Lastly, the study will inform the development of participatory interventions in comparable industries where low-wage workers experience both social and physical hazardous working conditions.

## Supporting information

S1 Checklist SPIRIT 2013 checklist: Recommended items to address in a clinical trial protocol and related documents*. (DOC)

S1 File (PDF)
