# Supplementary material for: The Fulfillment Center Intervention Study: Protocol for a group-randomized control trial of a participatory workplace intervention
Source: PLoS One. 2024 Jul 18;19(7):e0305334. doi: 10.1371/journal.pone.0305334 (PMC11257368; doi:10.1371/journal.pone.0305334)
Supplement: S1 File — (PDF) [file pone.0305334.s002.pdf]

Protocol Summary # 2008000224

Protocol Overview

Title:	**Fulfillment Center Intervention Study**		
Principal Investigator:	**Kelly, Erin L.**	Protocol Status:	**Active - Open to Enrollment**
Protocol Type:	**Comprehensive Review**	Lead Unit:	**Sloan School of Management**
Risk Level:	**No greater than minimal risk**	FDA Risk Level:	
Approval Date:	**09-11-2020**	Expiration Date:	**09-06-2024**
Anticipated Start Date:	**09-01-2020**	Anticipated End Date:	**09-01-2024**
IRB Admin:	**Keohane, Michael J**	Submission Status:	

Purpose of Study:

This study is an extension of the protocol for "FIRM Work Scheduling Study" (COUHES #1811595483) and seeks to evaluate how work conditions affect workers' health and well-being, job attitudes and decisions on the job, and key organizational outcomes. This study is being carried out in partnership with a national retailer's e-commerce division. The study would center on a cluster randomized trial evaluation of changes in workplace policies and practices in order to understand their impact on workers, families, and the firm but also utilize multi-method data to gain a better understanding of the stressors and sources of resilience for this growing, but understudied population of low-wage workers.

The primary goal of this study is to implement and test the feasibility of a participatory, integrated workplace intervention in fulfillment centers. The focal intervention is the launch of Health and Well-being Committees (HaWCs) in randomized fulfillment centers. HaWCs will involve 10-14 workers and frontline supervisors, with a worker and manager co-chair. HaWCs will identify challenges to safety, health, and well-being spanning physical hazards, COVID-19 protocols as relevant, other conditions of work (e.g., schedules, pace of work), and policies (e.g., absenteeism, performance management). They will then design and implement mitigating solutions and track outcomes. As such, HaWCs have a broader scope than traditional safety committees and will actively solicit worker voice regarding policies and practices that are not labeled as health and safety policies. We plan to use a mixture of administrative data, survey data, and interview data in the study. Administrative data will be used to measure employee hours, performance, absenteeism, and exits from the firm. Data from employee surveys will measure the effect of the workplace changes on wellbeing, job satisfaction, and similar measures. Interview data will be used to evaluate the process of launching and implementing changes, as well as to understand employee experiences and acute stressors during the COVID-19 pandemic. The research team will also use the data described above to assess how variations in management practices and work conditions over time and across sites affect both workers and the firm. To give two examples, scheduling practices that vary over time and across sites will be associated with employee outcomes (e.g., absenteeism, turnover, and wellbeing) and firm outcome (e.g., productivity) and the demographic composition of the workforce will be evaluated as a potential predictor of turnover, wellbeing, workers' promotions, wage growth, and injuries. These and related research questions look beyond the focal intervention to further our understanding of work conditions that affect this population of low-wage workers, and to identify potential changes to workplace policies and practices that support those workers' well-being and key organizational outcomes.

Data collection will take place at FIRM (generally during paid work time). We expect to collect data using web surveys, phone interviews, and text messages for the immediate future with the possibility of fieldwork and in-person interviews if deemed safe. The research partnership involves a nondisclosure agreement that requires us not to use the organization's name. As per agreement with FIRM, after merging, we will strip any administrative data that they share of identifying information such that subjects cannot be identified, directly or through identifiers linked to the subjects, in our analysis and reports.

Study Details:

This study will follow a cluster-randomized trial research design. The FIRM consists of a network of approximately 21 sites (warehouses). The goal of this study is to implement and test the feasibility of a participatory, integrated workplace intervention in fulfillment centers. The focal intervention is the launch of Health and Well-being Committees (HaWCs) in randomized fulfillment centers. HaWCs will involve 10-14 workers and frontline supervisors, with a worker and manager co-chair. HaWCs will identify challenges to safety, health, and well-being spanning physical hazards, COVID-19 protocols as relevant, other conditions of work (e.g., schedules, pace of work), and policies (e.g., absenteeism, performance management). They will then design and implement mitigating solutions and track outcomes. As such, HaWCs have a broader scope than traditional safety committees and will actively solicit worker voice regarding policies and practices that are not labeled as health and safety policies. Sites will randomized to treatment or control (after stratification or matching). In c onsultation with FIRM, one site will be designated as a proof-of concept site and that site will not be included in randomization. In treatment sites, the entire workplace will experience the launch of Health and Well-being Committees with administrative and interview data capturing whether there is variation in how the proposed changes are actually implemented across departments and shifts. It is expected that all control sites will also receive treatment after the experimentation period.

Survey data will be collected at three time points: baseline, 6 months after the intervention introduction, and 12 months after the intervention introduction. The 15-20-minute survey will be delivered via quantitative data collection software, Qualtrics, and will be administered via digital devices, like mobile phones or tablets. Participants will be able to complete the survey on company time and will provide electronic consent to participate each time. The survey will be provided in English and Spanish. An English version of the survey is attached -- Note that the Spanish survey translation is in progress so will be submitted by addendum. Each individual will be given a unique key to access the survey. The current plan for fall 2020 is that researchers will not be onsite for data collection, in conjunction with current COVID-19 protocols. This will be re-evaluated later. There is the possibility that researchers would visit the proof-of-concept site and early treatment sites in the next six months, to help launch the workplace change by providing training to managers and employees. Those interactions would follow strict protocols with masking and distance, and may occur outside in parking lot or break areas adjacent to the fulfillment center.

At the beginning of the survey, there will be an explanation of the purpose of the survey. That explanation is correct but does not name the particular workplace changes that are being assessed, since the control sites will not receive that intervention during the initial experimental period. Electronic consent will be obtained and the participant will receive a copy of the signed consent form via email, or will be given a physical copy if they do not have an email. Should participants have any questions or wish to discuss the research with a study team member prior to granting consent, the contact information will be included in the consent form.

Survey questions will address research outcomes of interest, such as: subjective well-being, schedule control, work/life conflict, culture of safety, trust in management, job control, stress, health and safety, work during COVID-19, worker voice, and job satisfaction. Demographic information (e.g. race/ethnicity, gender, and age) will also be obtained. At the 6-month and 12-month survey, those participants in treatment sites

will be asked about their awareness and approval of the committee. At the end of the survey, participants will be asked to voluntarily provide personal contact information (e.g. cell phone number) in order to: a) share findings with them even if they leave the FIRM and b) to potentially conduct follow-up surveys after they leave for another job or quit for other reasons. We may also ask for the co ntact information of one or two family members or friends should we be unable to contact the participant directly during follow-up. Providing this contact information will be explicitly voluntary.

A random subsample of individuals may be identified to receive additional communication about the workplace intervention to test whether these nudges affect knowledge of the changes in policies and practice and study outcomes.

We plan to engage a subset of employees and managers via phone, text, or a ethnographic data collection software, such as Indeemo, with contacts initiated up to a maximum of once per week in the first three months of the workplace change. This subset would be members of the Health   Wellbeing Committee or, with the scheduling intervention, managers and employees who expressed interest in telling us how it was working for them in the meeting introducing the new process. Consent for this type of data collection will be gained separately. An example of this consent form is attached as warehouse_study_consent_check-in.docx.

The plan for fall 2020 is that researchers will not be onsite to conduct the interview in conjunction with current COVID-19 protocols. As such, the qualitative interviews will be be conducted via phone or Zoom in a private room at the workplace, unless the respondent prefers to do the remote interview off-site. We aim for these interviews to be 1-on-1. Each interview should last 30 minutes to an hour. The remote nature of the interviews will be re-evaluated later.

At the start of the interview, we will explain the purpose of the interview and then walk through our consent form, which is attached. Once we have obtained consent, we will follow a semi-structured interview guide, an example of which is attached. Interview topics would include worker perspectives on the work environment during the initial COVID-19 period, how the participant responded or is responding to policy changes at the FIRM during this time (e.g., loosening attendance policies), whether work conditions have changed, feedback on the intervention as it is rolled out, and any pressing concerns they may have. Participants will not have to answer any questions they do not want to answer, and they may terminate the interview at any time. With consent, recording of the interviews will take place, either via Zoom for remote interviews or via an MP3 audio recording device if the interview takes place in-person.

In order to understand the process of how the intervention is launched and implemented, we will conduct check-in interviews with employees involved in or knowledgeable about the committee 2, 4, 6, 8, 10, 18, and 24 months after launch. The consent form for the interviews is attached. There are no incentives planned for this activity, but all interviews will take place during the regularly scheduled work day on paid time. We will also conduct interviews with the fulfillment center managers at 6, 12, and 30 months after launch. The consent form and interview questions are attached. Similarly to the check-ins with committee leaders, we will not be providing incentives for these interviews but they will take place during regularly scheduled work hours. This protocol also integrates the previous FIRM Work Scheduling Study (#1811595483), which involved collecting de-identified data and closed as a part of the the COUHES transition for old exempt studies. The previous study focused on

company data including HR, scheduling data, and productivity data that is provided to us directly and had different outcomes at the center (e.g., turnover, movement within the firm). By integrating the previous FIRM Work Scheduling Study, this study includes the data set provided by the FIRM.

Personal Information

**Name**	**Role**	**Study Role**	**Obtained Consent**	**Affiliation**
Rahmandad, Hazhir	Co-Investigator	Data Analysis	Y	Faculty
Wang, Molin	Co-Investigator	Research design and data analysis	N	Non-Faculty with PI Status
Kubzansky, Laura	Co-Investigator	Research design and data analysis	N	Non-Faculty with PI Status
Berkman, Lisa	Co-Investigator	data analysis	N	Non-Faculty with PI Status
Kelly, Erin L.	Principal Investigator		Y	Non-Faculty with PI Status
Lovejoy, Megan	Study Personnel	data analysis	Y	Non-Faculty with PI Status
Siebach, Kirsten F	Study Personnel	Program management	Y	Non-Faculty with PI Status
Yadama, Aishwarya P.	Study Personnel	Data Analysis	N	MIT Affiliate
Willison, Morgan	Study Personnel	Undergraduate Research Assistant	N	Student Investigator
Cho, Isabella	Study Personnel	Undergraduate research assistant	N	Student Investigator
Tronco, Katrina	Study Personnel	Research Assistant	N	Student Investigator

DeHorn, Grace Kathleen	Study Personnel	Project management and survey administration	Y	MIT Employee
Diaz-Linhart, Yaminette	Study Personnel	Data Analysis	Y	MIT Employee
Wagner, Gregory	Co-Investigator		N	Non-MIT Affiliate
Peters, Susan	Co-Investigator		N	Non-MIT Affiliate
Gunderson, Daniel	Co-Investigator		N	Non-MIT Affiliate
Langworthy, Ben	Co-Investigator	Data analysis	N	Non-MIT Affiliate
Fredericks, Michelle	Co-Investigator	Data Analyst	N	Non-MIT Affiliate
Javadi, Dena	Study Personnel		N	Non-MIT Affiliate
Kowalski, Alexander Marion	Study Personnel	Data analysis	Y	Non-MIT Affiliate

Protocol Units

Name	Type	Description
Sloan School of Management	Lead Unit	

Point of contact

Person Name	Role
Paulauskaite, Ieva	Correspondence Administrator

Funding Source

Source	Account	Funding Source	Name	Title
Sponsor	000310	000310	National Institute for Occupational Safety & Health	

Award	6941277	029622-00001	Washington Center for Equitable Growth	
Other		Good Companies, Good Jobs Initiative and Health Systems Initiative		MIT-Sloan providing funding

Subjects

Type	Minimum age	Maximum Age	Count
Adult Subjects	18	70	5000

Engaged Institutions

Name	Point of Contact	Approval Date	Expiration Date
Cornell University	Ceded to MIT		
Massachusetts Institute of Technology	COUHES		

Additional Forms

P1. Study Overview (Complete)   Updated By DeHorn, Grace Kathleen on 12/20/2022

Q. S.1: Describe the purpose of the research.

This study is an extension of the protocol for "FIRM Work Scheduling Study" (COUHES #1811595483) and seeks to evaluate how work conditions affect workers' health and well-being, job attitudes and decisions on the job, and key organizational outcomes. This study is being carried out in partnership with a national retailer's e-commerce division. The study would center on a cluster randomized trial evaluation of changes in workplace policies and practices in order to understand their impact on workers, families, and the firm but also utilize multi-method data to gain a better understanding of the stressors and sources of resilience for this growing, but understudied population of low-wage workers.

Q. S.2: What are the aims and hypotheses for this study?

The goal of this study is to implement and test the feasibility of a participatory, integrated workplace intervention in fulfillment centers. The focal intervention is the launch of Health and Well-being Committees (HaWCs) in randomized fulfillment centers. HaWCs will involve 10-14 workers and frontline supervisors, with a worker and manager co-chair. HaWCs will identify challenges to safety, health, and well-being spanning physical hazards, COVID-19 protocols as relevant, other conditions of work (e.g., schedules, pace of work), and policies (e.g., absenteeism, performance management). They will then design and implement mitigating solutions and track outcomes. As such, HaWCs have a broader scope than traditional safety committees and will actively solicit worker voice regarding policies and practices that are not labeled as health and safety policies. We plan to use a mixture of administrative data, survey data, and interview data in the study. Administrative data will be used to measure employee hours, performance, absenteeism, and exits from the firm. Data from employee surveys will measure the effect of the workplace changes on wellbeing, job satisfaction, and similar measures. Interview data will be used to evaluate the process of launching and implementing changes, as well as to understand employee experiences and acute stressors during the COVID-19 pandemic.

Q. S3: Describe your study endpoints. Scientific endpoints are outcomes defined before the study begins to determine whether the objectives of the study have been met and to draw conclusions from the data.

We expect to collect data using web surveys, phone interviews, and text messages for the immediate future alongside fieldwork and in-person interviews.

Q. A. Does the research involve collection of data or receipt of data from an international location(s)?

○ Yes

◉ No

Q. B. Is the project in, related to, or funded by a person or entity from China (including Hong Kong), Russia or Saudi Arabia?

- ○ Yes
- ◉ No

Q. C. Does the research involve collection of data from domestic locations? Excluding MIT campus.

- ◉ Yes
- ○ No

Q. C.1: List domestic study location(s):
FIRM warehouse in the United States

Q. C.2: Does the research involve collection of data online or in the digital space?

- ◉ Yes
- ○ No

Q. D. Does the research involve any collection of data on the MIT campus?

- ○ Yes
- ◉ No

Q. E. Is the study conducted in another language besides English?

- ◉ Yes
- ○ No

Q. E.1: List the language(s) below. Investigators must include translated documents and a completed Attestation Form with their submission.
Spanish. Translation and attestation was attached.

Q. F. Does the research involve the use of a drug or biological product?

- ○ Yes
- ◉ No

Q. G: Does the research involve the use of a device?

- ○ Yes
- ◉ No

Q. H: Is the research federally funded?

- ◉ Yes
- ○ No

Q. H.1: List the federal funding agency (ies) below.

National Institute for Occupational Safety & Health

Q. H.2: Has this research been issued an NIH Certificate of Confidentiality (CoC)?

- ○ Yes
- ◉ No

Q. H.3: Are participants prospectively assigned to one or more interventions (which may include a placebo or control)?

- ◉ Yes
- ○ No

Q. H.3.a: Is the research evaluating one or more interventions on human subjects' biomedical, behavioral status or quality of life?

- ◉ I will review the requirements.
- ○ Yes
- ○ No

Q. I. Are you collecting or using biospecimens that are identifiable to an individual as part of the research?

- ○ Yes
- ◉ No

Q. J. Will radiation or radioactive materials be used?

○ Yes

◉ No

Q. J.2: Will any type of laser be used?

○ Yes

◉ No

Q. J.3: Are subjects asked to adhere to a special diet?

○ Yes

◉ No

Data Classification (Complete) Updated By DeHorn, Grace Kathleen on 05/26/2023

Q. Q1- Will you be collecting, transmitting, analyzing, storing or otherwise handling data or information that either: directly identifies individuals, or with respect to which there is a reasonable basis to believe that the information or data could be used to identify individuals? This includes but is not limited to data containing personal identifiers, coded data with or without access to identifiers, audio and/or video recording, and storage of signed consent forms (both paper and electronic).

○ Yes

◉ No

Q. Q2- Will you be collecting, transmitting, analyzing, storing, or otherwise handling data or information that is protected by Federal, State or other laws or regulations?

☐ Data from EU/EEA(European Economic Area) and/or UK that is subject to General Data Protection Regulation (GDPR) or UK Data Protection Act

☐ Student educational records - Family Educational Rights and Privacy Act (FERPA)

☐ Protected health Information and/or medical records subject to the Health Insurance Portability and Accountability Act (HIPAA)

☐ Personally identifiable information (SSN, passport, etc.) - including, residents of the Commonwealth of Massachusetts - the Massachusetts Data Security Act

☐ Other

☑ No

Q. Q3- Will you be collecting, transmitting, analyzing, storing or otherwise handling any of the following?

- ☐ National Security Information
- ☐ Information regarding illegal activities
- ☐ Financial records
- ☐ Genetic Information
- ☑ Employment records
- ☐ Sexual preference
- ☑ Signed consent forms, contact information for recruitment or compensation
- ☐ De-identified data from public websites
- ☐ Anonymous specimens that are publicly available
- ☐ Not collecting data with any sensitivity at all (data is anonymous or de-identified without access to a linking code; AND, nothing in the data set could reasonably place the subject at risk of criminal or civil liability or be damaging to the subjects' financial standing, employability, educational advancement, or reputation. Please note: de-identified or anonymous Protected Health Information do NOT apply here).
- ☐ Other identifiable data
- ☐ Other non-identifiable data with uncertain sensitivity
- ☐ Other

Q. Q4- Will you be receiving data under a Data use Agreement (executed through RAS or OSATT) from a third party ?

- ○ Yes
- ◉ No

Q. Q5- What will happen to the data when the study is completed?

At the end of the study, data will be stored on a secure, encrypted MIT server for the Principal Investigator's projects. Deidentified data will be kept on this server for at least 5 years after the end of the study.

Survey data will be available through the restricted access process described below no later than the acceptance for publication of the main findings from the final dataset. Those who wish to use the data will need to complete an application with their affiliate institution and Institutional Review Board and provide a brief description of their project. The data sharing agreement will state that a) the data access will last for one year, with the opportunity to extend on a case-by-case basis, b) those who use the datasets will do so only for research or statistical purposes and not for investigation of the research subjects, c) published data will include the appropriate acknowledgement as stated in the data documentation, and d) the data will not be distributed to anyone other than the approved researcher.

Q. Q6- Can data acquired in the study affect a subjects relationship with others individuals (e.g. employee-supervisor, patient-physician, student-teacher, family relationships)?

No. Employee data will not be shared with supervisors, as described above, and any analysis shared with the FIRM will involve aggregate data or de-identified quotes with care taken to avoid inadvertent identification of the respondent.

Q. Does the research involve collection of data from an international location(s)?

- ○ Yes
- ◉ No

Q. Does the research involve collection of data from domestic locations? Excluding MIT campus.

- ◉ Yes
- ○ No

Q. List the domestic study location(s):
FIRM warehouse in the United States

Q. Does the research involve collection of data on the MIT campus?

- ○ Yes
- ◉ No

Q. Is the study conducted in another language besides English?

- ◉ Yes
- ○ No

Q. List the language(s) below. Investigators must include translated documents and a completed Attestation Form with their submission.
Spanish. Translation and attestation was attached.

Q. Does the research involve the use of a drug or biological product?

- ○ Yes
- ◉ No

Q. Does the research involve the use of a device?

- ○ Yes
- ◉ No

Q. Are participants assigned to one or more arms (e.g., intervention, placebo, or other control) of the research?

- (●) Yes
- ( ) No

Q. Is the research designed to evaluate the effect of manipulation of the participant or their environment for the purpose of one or more health-related or behavioral processes?

- (●) Yes
- ( ) No

Q. Is the research evaluating one or more interventions on human subjects' biomedical, behavioral status or quality of life?

- (●) Yes
- ( ) No

Q. Are you collecting or using biospecimens that are identifiable to an individual as part of the research?

- ( ) Yes
- (●) No

Q. Will radiation or radioactive materials be used?

- ( ) Yes
- (●) No

Q. Will any type of laser be used?

- ( ) Yes
- (●) No

Q. Are subjects asked to adhere to a special diet?

- ( ) Yes
- (●) No

Q. Is the project in, related to, or funded by a person or entity from China (including Hong Kong), Russia, or Saudi Arabia?

- ○ Yes
- ◉ No

INACTIVE - P2. Participant Information (Complete) Updated By Siebach, Kirsten F on 06/13/2022

Q. What are the criteria for inclusion and exclusion?
All workers at the warehouses are eligible for inclusion into the study

Q. Are any inclusion or exclusion criteria based on age, gender, or race/ethnic origin?
NA

Q. Explain the inclusion of any vulnerable population(s) and reason for their inclusion.
NA

Q. Is there a relationship between the subjects and investigator(s)? Including parent and child, doctor and patient, supervisor and employee/student, instructor and student. For research involving MIT students and lab members, see: http://couhes.mit.edu/guidelines/mit-students-and-lab-members-subjects.

- ○ Yes
- ◉ No

Q. Are you recruiting subjects?

- ◉ Yes
- ○ No

Q. Describe the recruitment procedures and the methods that will be used to identify and recruit subjects. Upload recruitment document(s) in the Attachments tab.
Study components will be introduced as part of regular workplace meetings and communications strategies. For example, departments have "daily huddle" meetings (which are now currently distanced with announcements and questions via megaphone) and the survey and interviews will be announced there. We will have posted materials and are likely to have a flyer for workers to pick up as they enter or leave. There is a possibility that we would have a short introduction video from the research team that may be played in a break room or during a meeting. Once an intervention direction has been decided and the communications drafted, we will submit communications to COUHES for approval, including posters for common spaces and a letter inviting each employee to participate. Though researchers will not be onsite to give the announcement, all printed materials (including talking points for managers) and video will explain that a survey is being conducted to learn more about workersÃ¢â¬â¢ perspectives on their jobs and how those perspectives change or donÃ¢â¬â¢t change in the future. Supervisors will explain that the survey is confidential, voluntary, approved by FIRM managers, and will be taken on company time. If an individual completes the survey, they will receive a $10 check in the mail. Additionally, they will be entered into a raffle for one of the four gift cards, with a (1) $150 gift card, a (1) $50 gift card and two (2) $25 gift cards given to randomly chosen survey participants in each site. This gift card protocol will be repeated for the two follow-up surveys, with all employees who are currently working at the fulfillment center eligible for the later survey waves. There will also be a building-level incentive

SEE COMPLETE DESCRIPTION IN THE APPLICATION UNDER ATTACHMENTS.

Q. Are you obtaining informed consent?

- (●) Yes
- ( ) No

Q. Upload consent forms in the Attachments tab.

- (●) I will upload the required consent forms.

Q. Does the study involve a pre-existing data set?

- ( ) Yes
- (●) No

Q. Are you requesting a waiver of consent?

- ( ) Yes
- (●) No

Q. Are subjects compensated?

- (●) Yes
- ( ) No

Q. Describe all plans to compensate subjects.

Each individual who submits responses to the survey will be asked to provide their home address so that they can be sent a $10 gift card in the mail. Each site will be given a (1) $150 gift card, a (1) $50 gift card and two (2) $25 gift cards for each survey wave. If an individual completes the survey, they will be entered into a raffle for one of the three gift cards. The recipients of the gift cards will be randomly chosen from survey participants. There will also be a building-level incentive. Participants who complete an interview to provide feedback on the planned workplace intervention will be offered a $25 gift card.

Q. Will the subjects be reimbursed for travel and expenses?

- ( ) Yes
- (●) No

INACTIVE - P3 Risks, Benefits, & Data Security (Complete) Updated By Siebach, Kirsten F on 06/13/2022

Q. What are the risks / discomforts associated with each intervention or procedure in the study?

We do not anticipate any risks to participating in this study other than those faced in day-to-day work life.

Q. What procedures will be in place to prevent / minimize potential risks or discomfort?

All communication and consent will emphasize that participation is completely voluntary and information will be confidential. Workers will not face repercussions for participating or not participating. At any point, they can choose to no longer participate or to skip questions or stop recording (in interviews).

Q. What potential benefits may subjects receive from participating in the study?

Based on exploratory interviews from our previously approved protocol (COUHES #1811595483), it is clear that fatigue, frustrations with unpredictable schedules, and acute work-life conflicts stemming from long hours and inflexible schedules contribute to increased stress of workers in this environment. The COVID-19 pandemic has only heightened this stress. The intervention we intend to introduce will seek to provide some relief from stresses at work with the expectation that this will improve overall well-being of individuals, both inside and outside of work. Additionally, we expect that these workers will appreciate being able to share their perspective on their work and its relationship to their health, wellbeing, personal life, and job attitudes.

Q. What potential benefits can society expect from the study?

Warehouse work is a growing industry but little research has been done to understand the work experiences and well-being of warehouse workers. This study will lead to needed insight into this type of work, as well as test new ideas to improve employee health and well-being and support or improve productivity.

Q. What follow-up efforts will be made to detect any harm to subjects, and how will COUHES be kept informed?

Should there be unexpected adverse consequences in the study, such as retaliation for participating or not participating, participants can reach out directly to the research staff through the contact information provided on consent forms and recruitment posters or flyers. Additionally, they could report to their supervisors or to other managers (e.g., HR manager at that site) if they chose. The research team will regularly ask manager contacts for any concerns that have arisen about the study or the workplace intervention.

Q. Will information about the research purpose and design be withheld from subjects?

○ Yes

◉ No

Q. How will data be collected?

Survey data will be gathered and managed using MIT Qualitrics and will be uploaded to the secure server

Q. Will you be receiving data under a Data Use Agreement (DUA) or Memorandum of Understanding (MOU) from a third party?

○ Yes

◉ No

Q. Is there audio and/or video recording?

◉ Yes

○ No

Q. Explain how audio and/or video data is recorded and how the data is kept secure? Indicate if and when raw data files will be destroyed.

After gaining consent from the participant, we will record the remote interview audio using the "record meeting" feature on Zoom. If interviews occur in-person, we will record the audio using an audio recording device. Recording is voluntary. Interview audio will be transcribed and also uploaded to a secure server.

Q. How is the data stored and how will it be kept secure?

Data will be stored on secure, password protected, encrypted servers. All data, notes, recordings, transcripts, and FIRM documents will be stored securely, electronically until the project is complete. Physical backups may be stored in a locked office (E62-367). Interview recordings may be transcribed using a secure machine-based online transcriber. Data will be accessible only for authorized purposes and will be shared only with study personnel.

Q. What will happen to the data when the study is completed?

At the end of the study, data will be stored on a secure, encrypted MIT server for the Principal Investigator's projects. Deidentified data will be kept on this server for at least 5 years after the end of the study.

Survey data will be available through the restricted access process described below no later than the acceptance for publication of the main findings from the final dataset. Those who wish to use the data will need to complete an application with their affiliate institution and Institutional Review Board and provide a brief description of their project. The data sharing agreement will state that a) the data access will last for one year, with the opportunity to extend on a case-by-case basis, b) those who use the datasets will do so only for research or statistical purposes and not for investigation of the research subjects, c) published data will include the appropriate acknowledgement as stated in the data documentation, and d) the data will not be distributed to anyone other than the approved researcher.

Q. Do you plan to obtain, use or disclose identifiable health information through a covered entity in connection with the research?

- ○ Yes
- ◉ No

Q. Does the investigator, study personnel, or their Family have a financial interest in a company or other organization involved in this study?

- ○ Yes
- ◉ No

Q. Could the research reasonably appear to affect a company or other organization in which the investigator, study personnel, or their Family have a financial interest?

- ○ Yes
- ◉ No

Q. Receiving or using any data (e.g., proprietary data sets, data sets, confidential information) from a company or other entity organization in which the investigator, study personnel, or their Family have a financial interest

○ Yes

◉ No

Q. Receiving or using any materials (e.g., drugs, devices, biological agents, investigational medical devices) from a company or other entity organization in which the investigator, study personnel, or their Family have a financial interest.

○ Yes

◉ No

Q. Granting subawards to a company or other entity organization in which the investigator, study personnel, or their Family have a financial interest.

○ Yes

◉ No

Q. Making purchases from a company or other entity organization in which the investigator, study personnel, or their Family have a financial interest.

○ Yes

◉ No
